# MLH1 expression sensitises ovarian cancer cells to cell death mediated by XIAP inhibition

**DOI:** 10.1038/sj.bjc.6605180

**Published:** 2009-07-14

**Authors:** X Ding, A B Mohd, Z Huang, T Baba, M Q Bernardini, H K Lyerly, A Berchuck, S K Murphy, A B Buermeyer, G R Devi

**Affiliations:** 1 Department of Surgery, Duke University Medical Center, Durham, NC 27710, USA; 2 Department of Environmental and Molecular Toxicology, Oregon State University, Corvallis, OR 97333, USA; 3 Department of Obstetrics and Gynecology, Duke University Medical Center, Durham, NC 27710, USA; 4 Duke Comprehensive Cancer Center, Duke University Medical Center, Durham, NC 27710, USA

**Keywords:** OVCAR3, SKOV3, p53, Cisplatin, MMR, 6-thioguanine

## Abstract

**Background:**

The X-linked inhibitor of apoptosis protein (XIAP), an endogenous apoptosis suppressor, can determine the level of caspase accumulation and the resultant response to apoptosis-inducing agents such as cisplatin in epithelial ovarian cancer (EOC). In addition, the mismatch repair protein, hMLH1, has been linked to DNA damage-induced apoptosis by cisplatin by both p53-dependent and -independent mechanisms.

**Methods:**

In this study, hMLH1 expression was correlated with clinical response to platinum drugs and survival in advanced stage (III–IV) EOC patients. We then investigated whether MLH1 loss was a determinant in anti-apoptosis response to cisplatin mediated by XIAP in isogenic and established EOC cell lines with differential p53 status.

**Results:**

The percentage of cells undergoing cisplatin-induced cell killing was higher in MLH1-proficient cells than in MLH1-defective cells. In addition, the presence of wild-type hMLH1 or hMLH1 re-expression significantly increased sensitivity to 6-thioguanine, a MMR-dependent agent. Cell-death response to 6-thioguanine and cisplatin was associated with significant proteolysis of MLH1, with XIAP destabilisation and increased caspase-3 activity. The siRNA-mediated inhibition of XIAP increased MLH1 proteolysis and cell death in MLH1-proficient cells but not in MLH1-defective cells.

**Conclusion:**

These data suggest that XIAP inhibitors may prove to be an effective means of sensitising EOC to MLH1-dependent apoptosis.

A yet unmet need exists for novel therapeutic strategies to overcome resistance to chemotherapeutic treatment in epithelial ovarian cancer (EOC) with functionally inactivated p53. This subtype characterises 40–70% of ovarian cancers and renders a chemoresistant phenotype that is more difficult to overcome than that in p53 wild-type (wt) tumours (Lavarino *et al*, 2000; Canevari *et al*, 2006). Although p53 status has a leading function in determining the efficacy of apoptotic signalling, tumour cells including EOC can evade apoptosis using multiple mechanisms (Johnstone *et al*, 2002; Kaufmann and Vaux, 2003; Quintieri *et al*, 2007). The inhibitor of apoptosis protein (IAP) family has a significant function in apoptosis resistance because of the unique ability of most of the family members (IAP1, IAP2, Livin and XIAP) to inhibit both initiator- and effector caspases (Liston *et al*, 1996; Ambrosini *et al*, 1997). The X-linked inhibitor of apoptosis protein (XIAP) is considered to be one of the most potent IAPs and a versatile caspase inhibitor (Deveraux *et al*, 1997; Devi, 2004). Multiple studies in EOC (Sasaki *et al*, 2000; Asselin *et al*, 2001; Li *et al*, 2001; Mansouri *et al*, 2003; Yang *et al*, 2005) and other cancers (Amantana *et al*, 2004; Schimmer *et al*, 2006; Aird *et al*, 2008) have identified XIAP suppression to be a key mechanism in chemotherapy resistance. The acquisition of cisplatin resistance is associated with the ability of treated cells to upregulate XIAP expression. In addition, XIAP downregulation can induce apoptosis in chemoresistant EOC cells, although apparently only in cells that have a functionally active p53.

However, cancer cells with mutant p53 can undergo apoptosis in response to cisplatin by p53-independent mechanisms; indeed, in some EOC cell lines, the activity of caspase-3 is more predictive of apoptotic response than is the p53 status (Gong *et al*, 1999; Kolfschoten *et al*, 2002). Recently, hMLH1, a key DNA mismatch repair (MMR) protein, has been identified as a substrate of caspase-3-mediated proteolysis, resulting in the generation of a pro-apoptotic carboxyl-terminal product (Chen *et al*, 2004). The MLH1 is one of five proteins crucial to MMR function, the loss of which is associated with the acquisition of a mutator phenotype, including microsatellite instability (MSI) and predisposition to cancer (Jiricny, 2006). The MMR pathway reduces spontaneous mutation through the recognition and repair of DNA base–base mismatches and insertion–deletion loops generated during replication (Bellacosa, 2001; Kunkel and Erie, 2005; Iyer *et al*, 2006). In addition, MMR status is associated with cytotoxic cellular responses to various chemotherapeutics (Fink *et al*, 1998a, 1998b) because of the MMR-dependent activation of cell-cycle checkpoint and apoptosis signalling pathways (Stojic *et al*, 2004). A relationship between MMR status and sensitivity to cisplatin and platinum analogues has been widely reported (Aebi *et al*, 1996; Drummond *et al*, 1996; Brown *et al*, 1997; Fink *et al*, 1998b; Moreland *et al*, 1999; Massey *et al*, 2003; Helleman *et al*, 2006). Earlier studies using repair-defective variants of the A2780 (MLH1 proficient, wt-p53) ovarian cell line have shown that MLH1 restoration sensitises cells to DNA-damaging agents, although sensitivity to cisplatin remained significantly dependent on p53 function (Aquilina *et al*, 2000; Branch *et al*, 2000). Consistent with this, cisplatin-resistant ovarian cell lines have been observed to acquire an MSI phenotype and are defective in strand-specific MMR (Watanabe *et al*, 2001). Several reports link MLH1 status and clinical response (CR) in cancer patients (O'Brien and Brown, 2006). In contrast, some clinical reports (Scartozzi *et al*, 2003; Helleman *et al*, 2006) suggest no direct association between cisplatin response and MMR status in primary ovarian cancers.

In this study, MLH1 expression levels correlated with CR and increased survival among advanced stage EOC patients. Unselected ovarian cell lines, OVCAR3, which has wt MLH1 and MLH1-defective SKOV3, were compared with the A2780-MMR-defective variant, A2780-MNU1, in which hMHL1 was re-expressed by stable transfection (Aquilina *et al*, 2000). MLH1 proteolysis and XIAP destabilisation were observed in cisplatin response and 6-thioguanine (6-TG)-mediated apoptosis. In addition, MLH1 proteolysis increased active forms of caspase-3 and -9, and decreased cell viability was observed when XIAP was specifically inhibited using a siRNA in the MLH1-proficient and not in the MLH1-deficient ovarian cancer cells. Our study identifies a potential inverse correlation between MLH1 expression and XIAP anti-apoptotic activity in chemotherapy response in ovarian cancer cells.

## Materials and methods

### Cell culture and viability assay

The EOC cell lines were cultured in accordance with the manufacturer's recommended media (American Type Culture Collection, Manassas, VA, USA). A2780MNU1-MLH1 and A2780MNU1 cell lines were cultured according to earlier studies (Aquilina *et al*, 2000; Mohd *et al*, 2006). Cells at 50% confluence in a 96-well plate (Corning Incorporated, Corning, NY, USA) were treated with 0–200 *μ*mol l^−1^ cisplatin (Sigma Chemical Co., St Louis, MO, USA) for 24 h in a serum-containing medium. Cell were then incubated at 37°C for 2 h in a medium containing 0.5 g l^−1^ 3-(4,5-dimethylthiazol-2-yl)-2,5-diphenyltetrazolium bromide (MTT; Sigma Chemical Co.), and a proliferation assay was conducted as reported earlier (Amantana *et al*, 2004; Aird *et al*, 2008). In some experiments, cell viability was also determined by Trypan blue exclusion assay (Aird *et al*, 2008).

### Clonogenecity assay

Clonogenic survival of individual cultures after exposure to 6-TG was performed as described (Buermeyer *et al*, 1999). Cells were exposed to the indicated concentrations (0–10 *μ*M) of 6-TG diluted into growth media for 7–10 days, and surviving colonies (greater than 100 cells) were counted. The results for each dose are reported as the percentage of surviving colonies relative to mock-treated cultures, and represent the average (+/− standard deviations) of a minimum of 3–4 experiments.

### Western immunoblots

Cells were harvested and immediately lysed in an NP40 cell lysis buffer (Biosource, Carlsbad, CA, USA), subjected to SDS–PAGE under reducing conditions and transferred as reported earlier (Aird *et al*, 2008). The membranes were probed overnight with a primary antibody against XIAP (1 : 1000 dilution; BD Transduction Laboratories, Lexington, KY, USA), PKC-*δ* (1 : 1000; Cell Signaling Technology, Beverly, MA, USA), procaspase-9 (1 : 1000, Neomarker, Fremont, CA, USA), MLH1, PMS2 and MSH6 (1 : 500, BD Pharmingen, Lexington, KY, USA) at 4°C. Immunoreactive bands were visualised as reported earlier (Aird *et al*, 2008). *β*-Actin or GAPDH served as loading control by re-probing the same membrane with an antibody (1 : 4000; Calbiochem, La Jolla, CA, USA) or probed after first stripping the membrane in a stripping buffer (100 mmol l^−1^ 2-mercaptoethanol-2% SDS-62.5 mmol l^−1^ Tris-HCl (pH 6.7)) at 50°C for 30 min, followed by a washing and blocking procedure as described above.

### siRNA delivery

A total of 100 nM XIAP siRNA, GAPDH control siRNA (Cell Signaling) or control siRNA (5′-VUUCUCCGAACGUGUCACGU-3′, random sequence (Qiagen, Valencia, CA, USA) was transfected into cells as per manufacturer's instructions and earlier study (Aird *et al*, 2008) and incubated at 37°C for 48 h.

### Active caspase-3 detection

A PE-conjugated polyclonal active caspase-3 Ab apoptosis kit (BD Pharmingen) was used to quantify apoptosis by detecting the levels of active caspase-3, according to the manufacturer's directions. Cells were treated with vehicle, cisplatin (24 h) or with various siRNA (48 h). The cells were harvested and washed twice with cold PBS, then resuspended in Cytofix/Cytoperm solution at a concentration of 1 × 10^6^ cells per 0.5 ml and incubated for 20 min on ice. The cells were pelleted, washed, resuspended in 0.5 ml Perm/Wash Buffer and analysed with a FACScalibur flow cytometer (Becton Dickenson, San Jose, CA, USA).

### MLH1 expression analysis in ovarian cancer patients

Affymetrix U133A gene expression microarray data (Santa Clara, CA, USA) from which *hMLH1* expression levels were obtained have been described earlier (Berchuck *et al*, 2005). For this study, 54 of these patients with stage III or IV serous EOC, who received platinum-based chemotherapy, were included. Patients in this data set lived for <3 years (*N*=30) or for longer than 7 years (*N*=24) after diagnosis. Log-transformed gene expression values were calculated using robust multiarray analysis (Irizarry *et al*, 2003). The probe for *hMLH1* (202520_s_at) on the Affymetrix U133A genechip was used for analysis. Two-tailed, unpaired *t*-tests were used to compare the expression of *hMLH1* in patients on the basis of CR and survival.

### Statistical analysis

Statistical analyses were performed using GraphPad Prism 4.0 (La Jolla, CA, USA). Differences were considered significant at *P*<0.05. Data are expressed as mean±s.e.m.

## Results

### Elevated MLH1 correlates with ovarian cancer patient survival

**Figure 1:**
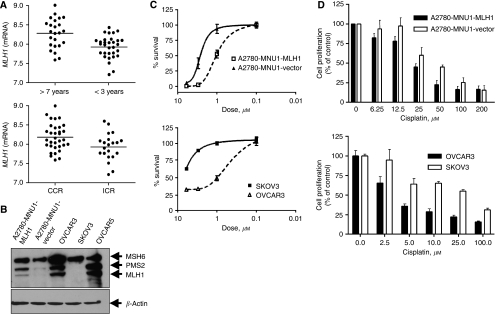
MLH1 expression and chemotherapy sensitivity in ovarian cancer cells and patients. (**A**) Correlation of *MLH1* mRNA expression in microarray analysis using log-transformed Robust Multiarray Analysis values (*y* axis) from 54 stage III or IV ovarian cancer patients with clinical response (top graph) and survival (bottom graph) as described in the text. CCR refers to complete clinical response and ICR refers to incomplete clinical response. (**B**) The cellular abundance of MLH1, MSH6 and PMS2 was determined by immunoblot analysis of whole-cell lysates prepared from MLH1-deficient (SKVO3 and A2780MNU1 vector, a cisplatin-resistant variant of the A2780 ovarian carcinoma cell line) and hMLH1-proficient (OVCAR3, OVCAR5 and A2780MNU1-MLH1) cells. The membranes were stripped and reprobed with *β*-actin antibody to ensure an equal protein loading. Arrows indicate 84 kDa MLH1, 96 kDa PMS2, 160 kDa MSH6 and 35 kDa *β*-actin bands. (**C**) 6-thioguanine clonogenic assay. Top graph. A2780-MNU1-MLH1 compared with the MLH1-defective A2780-MNU1vector. Bottom graph. OVCAR3, which has wild-type MLH1 compared with MLH1-defective SKOV3 cells. Cells were treated with varying amounts of 6-TG for 24 h and exposed to a drug-free medium for 8 days. Cell viability in **C** and **D** was determined by clonogenicity analysis. Values are mean±s.e.m. (*n*=3). (**D**) Cisplatin MTT proliferation assay. Cells as indicated in top and bottom graphs were treated with different concentrations of cisplatin for 24 h, with proliferation determined by MTT assay. Bars represent mean±s.e.m. (*n*=3, each experiment in triplicate).

To correlate *MLH1* expression with clinical outcome in patients with ovarian cancer, microarray expression data (as described in Materials and Methods section) were analysed for a total of 54 patients with advanced stage serous EOC, who had received either cisplatin or carboplatin as part of their primary chemotherapeutic treatment. Patients exhibiting a complete clinical response (CCR) (CA125 <20 U ml^−1^; CAT scan and office exam showing no evidence of disease, assessed 1 month after the patient's last cycle of chemotherapy) had higher levels of *MLH1* compared with patients with an incomplete clinical response (ICR) (*P*=0.01, 95% confidence interval, 0.057–0.45), as shown in Figure 1A, top panel. Patients with higher levels of *MLH1* also exhibited a survival advantage, with elevated levels of *MLH1* mRNA present in tumours from women who lived longer than 7 years after diagnosis, compared with women who lived for <3 years after diagnosis (*P*=0.0005, 95% confidence interval, 0.15–0.52), as shown in Figure 1A, bottom panel. Thus, the MLH1 status in this patient cohort was associated both with increased CR and with increased long-term patient survival. Although the results of our analysis of MLH1 in EOC patients were consistent with earlier reports associating MLH1 status with CR in ovarian cancer patients, it is clear that MLH1 is not the sole determinant, and that other factors must also influence patient response and survival, in particular apoptotic dysregulation.

### MLH1 expression and response to cisplatin and 6-TG

As published preclinical and clinical studies show that p53 status may not by itself predict cellular response to cisplatin, we investigated the apoptotic pathway engaged in response to MLH1-dependent signalling in a set of MLH1-proficient and MLH1-deficient EOC cells with an inactive or null p53 status. Two widely studied ovarian tumour cell lines – OVCAR3 (expressing wt hMLH1) and SKVO3 (deficient in endogenous MLH1) – were characterised along with A2780MNU1, an MLH1 and a p53-deficient clonal derivative of A2780. The parental A2780 is a well-characterised ovarian carcinoma cell line that is proficient in MMR and has an intact p53 response. Human MLH1 was re-expressed in A2780MNU1 by transfection to create a clonal cell derivative – A2780-MNUI-MLH1 – and the corresponding vector-only-transfected A2780-MNU1 vector lines. An immunoblot analysis of the MMR status (MLH1, PMS2, MSH6, key members of MMR family) (Figure 1B) reveals an MSH6 protein expression in all four cell lines irrespective of MLH1 status. The MLH1 as well as the PMS2 protein were expressed and accumulated in MLH1-positive cell lines (A2780MNU1-MLH1, OVCAR3 and OVCAR5), whereas no MLH1 and decreased PMS2 levels were detected in A2780MNU1 cells and SKOV3 cells, consistent with the role of MLH1 in stabilising PMS2.

A2780MNU1-MLH1, A2780-MNU1 vector, OVCAR3 and SKVO3 cells were evaluated for sensitivity to 6-TG, a chemotherapeutic purine nucleoside analogue, the primary mechanism of action of which is dependent on the presence of a functional DNA MMR system. The hMLH1 re-expression in A2780MNU1 cells significantly increased sensitivity to 6-TG compared with that in the MLH1-deficient A2780MNU1 vector, viability being determined by clonogenic survival (Figure 1C). The appearance of apoptotic cells was delayed and followed a G2/M phase of cell-cycle arrest (data not shown). Similarly, OVCAR3 cells, which have endogenous hMLH1, were highly sensitive to 6-TG compared with hMLH1-defective SKOV3, (Figure 1D), revealing that 6-TG response was dependent on MLH1 expression and was potentially p53 independent, as all the above four cell lines have non-functional p53.

The effect of wt MLH1 or MLH1 restoration on cisplatin sensitivity was characterised by treating the four cell lines with cisplatin using published dose ranges 0–200 *μ*M; MTT assay was used to determine growth and cell proliferation at 24 h. Data in Figure 1D identify OVCAR3 to be the most sensitive to cisplatin treatment, followed by A2780-MNU1-MLH1, the A2780-MNU1 vector and SKVO3. Rounding of cells as observed by microscopy (data not shown) and the measurement of the percentage of cells undergoing cisplatin-mediated cell death by Trypan blue cell exclusion assay at 24 h and 48 h showed decreased cell viability at a lower cisplatin concentration or at an earlier time point in hMLH1-proficient cells compared with that in hMLH1-defective counterparts (50% viability compared with control at 50 *μ*M in A2780MNU1-MLH1 *vs* 200 *μ*M in the A2780MNU1 vector, and 10 *μ*M in OVCAR3 at 48 h *vs* 100 *μ*M in SKOV3 at 48 h).

Overall, MLH1 was critical for 6-TG response, and a trend towards decreased cell viability in response to cisplatin was observed in MLH1-proficient cells.

### Destabilisation of XIAP and PKC-*δ* cleavage in cisplatin and 6-TG-responsive MLH1-proficient ovarian cancer cells

As cisplatin caused decreased cell viability in the MLH1-proficient cells tested, the expression of apoptotic- and anti-apoptotic proteins was conducted in attached and detached cisplatin-treated cell lysates at doses that showed significant detached cells and decreased cell viability at 24 h (50 and 200 *μ*M) for A2780MNU1-MLH1 and OVCAR3. This was compared with doses and time points on the basis of a 50% decrease in viability for MLH1-deficient cell lysates (200 *μ*M in the A2780MNU1 vector at 24 h and 100 *μ*M in SKOV3 at 48 h).

**Figure 2:**
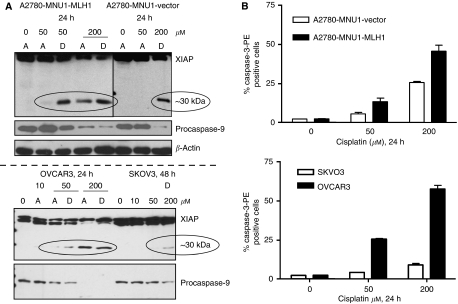
Analysis of XIAP expression in lysates from cisplatin-treated ovarian cells. (**A**) Immunoblot analysis of XIAP (55 kDa intact and 30 kDa proteolytic fragment) and procaspase-9 indicated. Top panel: comparison of MLH1-proficient A2780MNU1-MLH1 and MLH1-deficient A2780MNU1 vector; bottom panel: comparison of OVCAR3 (MLH1 positive) and SKVO3 (MLH1 deficient) ovarian cancer cells. A indicates adherent and D indicates detached in the immunoblots. (**B**) Graphical representation of relative cell numbers expressing active caspase-3 analysed by flow cytometric analysis using anti-caspase-3-PE antibody in the indicated cell lines treated with cisplatin at 24 h.

Immunoblot analysis (Figure 2A) of the expression of XIAP revealed a marked XIAP cleavage starting to occur in the detached cell population at 50 *μ*M and in both attached and detached cell populations at 200 *μ*M in cisplatin-treated A2780MNU1-MLH1 cells and OVCAR3. In contrast, XIAP cleavage was only observed in the detached 200 *μ*M concentration of cell lysates of the A2780-MNU1 vector at 24 h and at 200 *μ*M in SKOV3 at 48 h. Further, decrease/proteolysis of XIAP corresponded with a decrease in inactive procaspase-9, consistent with the ability of intact XIAP to suppress apoptosis by binding procaspase-9 and not allowing enzymatic cleavage to its active form. Apoptotic cell populations were also quantified using antibodies to active caspase-3-PE at 24 h by flow cytometry, which revealed an increase in the number of cells positive for the active caspase-3 protein in the A2780MNU1-MLH1 (Figure 2B, top graph) and OVCAR3 (Figure 2B, bottom graph) cells treated with cisplatin compared with that in the MLH-1-deficient cell lines in each graph panel.

**Figure 3:**
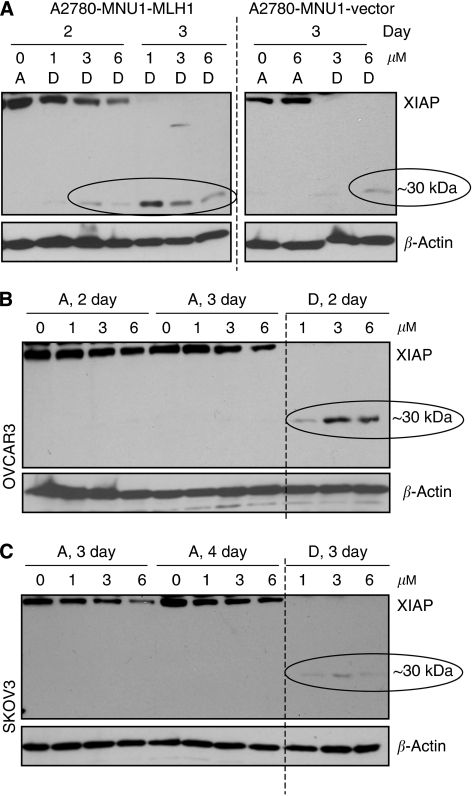
Analysis of XIAP expression in lysates from 6-TG-treated ovarian cells: immunoblot analysis of XIAP (55 kDa intact and 30 kDa proteolytic fragment) indicated in (**A**) A2780-MNU1-MLH-1 and A2780-MNU1 vector; (**B**) OVCAR3; and (**C**) SKVO3. The immunoblots were stripped and probed for total *β*-actin to show equal protein loading. A indicates adherent and D indicates detached in the immunoblots.

Similar to cisplatin response, XIAP proteolysis (Figure 3) corresponded with sensitivity to 6-TG (the earlier Figure 1 shows less than 50% cell survival starting at 3 μM 6-TG dose) in MLH1-proficient A2780MNU1-MLH1 and OVCAR3 cells. In MLH1-proficient cells (Figure 3A, left panel and 3B), the 30 kDa proteolysed fragment of XIAP started appearing in the detached cells after 2 days of treatment with 3 *μ*M 6-TG concentration, which increased with dose and time. In contrast, the appearance of the XIAP cleavage product was observed 3–4 days after treatment with a higher 6-TG (6 *μ*M) concentration in MLH1-deficient cells (Figure 3A, right panel; Figure 3C).

**Figure 4:**
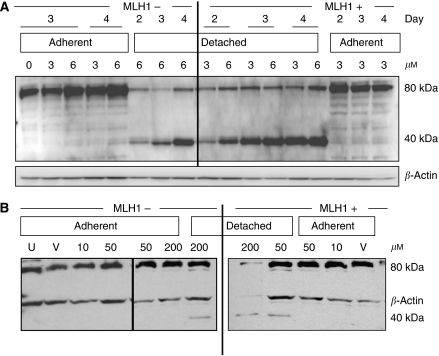
Immunblots of PKC-*δ* (80 kDa) and its cleavage product (40 kDa) in 6-TG (**A**)-treated cell lysates at indicated concentrations and time points and in cisplatin (**B**)-treated lysates at 24 h at indicated concentrations.

Furthermore, in MLH1-proficient cells, proteolysis of PKC-*δ* (Figure 4), a caspase-3 substrate and marker of apoptotic cell death, was identified in the lysates of detached cell populations at both 3 and 6 *μ*M 6-TG treatment concentrations on days 2, 3 and 4 in MLH1-proficient cells compared with a 6 *μ*M 6-TG concentration in MLH1-deficient cell lysates (Figure 4A). Similarly, the 40 kDa PKC-*δ* proteolytic fragment was observed in cell lysates of cisplatin-treated A7280-MNU1-MLH1-detached cells (50 and 200 *μ*M) compared with the appearance of the 40 kDa fragment at 200 *μ*M detached cells in A2780-MNU1 vector cells (Figure 4A). *β-*Actin is shown with each blot as a loading control.

The combined results show that MLH1-proficient cells are more sensitive to a cisplatin-mediated decrease in cell viability, corresponding to a significant destabilisation of XIAP, increased caspase-3 and -9 and PKC-*δ* proteolysis.

### MLH1 cleavage in cisplatin and 6-TG-induced apoptosis in ovarian cancer cells

**Figure 5:**
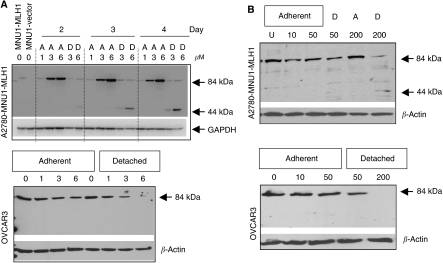
MLH1 expression in A2780-MNU1-MLH1 and OVCAR3. (**A**) Arrows indicate the intact (84 kDa) and/or proteolytic fragment (44 kDa) of MLH1 in lysates of adherent and detached cell lysates grown in the presence of 6-TG (1, 3, 6 *μ*M) for 2, 3 or 4 days as indicated. (**B**) MLH1 expression in lysates of cisplatin (10, 50, 200 *μ*M)-treated cells at 24 h. A indicates adherent and D indicates detached in all the above immunoblots.

To determine the effect of apoptosis signalling on MLH1 expression, MLH1 immunoblot analyses were conducted for lysates of A2780-MNU1-MLH1 and OVCAR3 cells treated with 6-TG (Figure 5A) and cisplatin (Figure 5B). Data show a decrease in MLH1 expression in a dose- and time-dependent manner after 6-TG and cisplatin treatment in the detached cell lysates of the two MLH1-proficient cell lines. Further, a significant proteolysis of 84 kDa intact MLH1 to approximately 44 kDa fragment was also observed in A2780-MNU1-MLH1 in a dose- and time-dependent manner (Figure 5A and B, top panels). The MLH1 cleavage fragment was evident in cells that had started detaching than in adherent cells. The GAPDH or *β*-actin are shown as loading controls. The extent to which this fragment contributes to the MLH1-dependent signalling of apoptosis is currently under investigation.

### XIAP inhibition selectively decreases cell viability in MLH1-proficient ovarian cancer cells by MLH1 proteolysis

**Figure 6:**
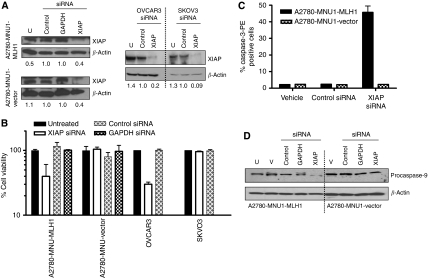
Effect of XIAP siRNA in ovarian cancer cells. Cells were treated with indicated siRNA for 48 h. (**A**) Immunoblot analysis of the XIAP protein (55 kDa) expression in untreated, XIAP siRNA- and control siRNA-treated cell lysates. (**B**) Cell viability was monitored by Trypan blue exclusion assay (*n*=3 with quadruplicates in each *n*) and expressed as percentage untreated. The *x* axis shows various siRNA treatment and none indicates transfection reagent alone. (**C**) Graphical representation of relative cell numbers expressing active caspase-3 analysed by flow cytometric analysis using anti-caspase-3-PE antibody. (**D**) Immunoblot analysis of procaspase-9 expression.

Cisplatin sensitivity in ovarian cancer has been correlated with inhibition of XIAP, consistent with the observations in this study (Figure 2). In addition, XIAP destabilization in cisplatin- and 6-TG-sensitive MLH1-proficient cells corresponded with MLH1 decrease and/or increased proteolysis. (Figure 5). To understand the relationship between MLH1 status and cell-death response in the absence of functional p53, XIAP was specifically inhibited using a siRNA strategy in the panel of MLH1-proficient and -defective EOC cells. The XIAP protein abundance was decreased in the four cell lines tested 48 h after XIAP siRNA transfection compared with that in siRNA controls (Figure 6A). Data in Figure 6B show that XIAP protein downregulation in the hMLH1-defective and p53-mutant A2780MNU1 vector and SKOV3 cells had no effect on cell viability as measured by the Trypan blue exclusion assay. This result is consistent with an earlier report (Sasaki *et al*, 2000) suggesting that functional p53 is necessary for cells to respond to XIAP inhibition. In contrast, specific XIAP downregulation in p53-defective, MLH1-proficient cells (A2780MNU1-MLH1 and OVCAR3) caused significant reductions in cell viability; survival of EOC cells was reduced by ∼75% compared with that of untreated cells or of cells treated with control siRNA.

Apoptotic cell populations were quantified using antibodies to active caspase-3-PE by flow cytometry (Figure 6C), which revealed a significant increase in the number of cells positive for active caspase-3 protein in the A2780MNU1-MLH1 cells treated with XIAP siRNA compared with the A2780MNU1 vector. Furthermore, the immunoblot analysis (Figure 6D) of protein lysates of XIAP siRNA-treated MLH1-proficient cells (A2780-MNU1-MLH1 shown here) showed decreased inactive procaspase-9 levels. The combined treatment of XIAP siRNA with cisplatin did not result in greatly enhanced (i.e., synergistic) cytotoxic responses relative to either single exposure under these conditions (data not shown), indicating a need for testing other XIAP inhibitors and/or dosing regimens with cisplatin. Alternatively, a potent synergistic or additive effect of cisplatin and XIAP inhibition expression may require the presence of both wt-p53 and MLH1. Together, these data suggest that, similar to cisplatin-induced apoptosis, cell death mediated by XIAP inhibition in EOC cells with inactive p53 seems to require the presence of MLH1.

## Discussion

To understand the influence of MLH1 status on CR and long-term survival of advanced EOC patients, we have investigated the apoptotic signalling pathways engaged in p53-deficient ovarian cancer cells in response to chemotherapeutic agents, cisplatin and 6-TG, and have identified a novel link between the expression of the mismatch repair protein MLH1 and the stability of XIAP, an anti-apoptotic protein and caspase inhibitor. The XIAP inhibition and proteolysis were selectively observed in MLH1-proficient cells undergoing MLH1-dependent apoptosis induced in response to both 6-TG and cisplatin. In addition, proteolysis of MLH1 was observed in cells undergoing MLH1-dependent apoptosis. The MLH1 proteolysis corresponded with increased active forms of caspase-3 and -9 and significantly decreased cell viability. Our results suggest that overcoming XIAP-mediated inhibition of apoptosis is an important signalling event in the MLH1-dependent induction of apoptosis in p53-deficient ovarian cancer cells. This functional link between MLH1 and XIAP suggests that XIAP status may influence the effectiveness of MLH1-dependent responses to chemotherapy and that an inhibition of XIAP, in particular in MLH1-proficient cancers, may improve the CR of EOC patients to chemotherapy.

A number of studies *in vitro* (Aebi *et al*, 1996; Brown *et al*, 1997; Fink *et al*, 1998b; O'Brien and Brown, 2006) have reported a correlation between MMR deficiency and platinum resistance. Indeed, studies link MMR deficiency with reduced CR to platinum-based chemotherapy (Fink *et al*, 1998b; Strathdee *et al*, 2001; Gifford *et al*, 2004). In this study, microarray expression data from 54 patients with advanced stage serous EOC, who had received either cisplatin or carboplatin as part of their primary chemotherapeutic treatment, revealed a correlation between a high MLH1 expression in the patients' tumours and increased CR and survival. This supports the general concept that DNA damage caused by platinum drugs is recognised by MMR proteins leading to the induction of apoptosis. In contrast, in cancer cells with MLH1 deficiency, DNA damage is not sensed by repair proteins, resulting in a reduced apoptotic response and increased cisplatin resistance. However, not all studies in EOC patients have shown a direct relationship between MMR deficiency due to decreased MLH1 and cisplatin resistance (Scartozzi *et al*, 2003; Helleman *et al*, 2006). Interestingly, Scartozzi *et al* (2003) reported that MLH1 loss in a small number of stage III–IV EOC patient tumours correlated with improved survival. The tumour set from this study comprised only 44% serous EOC analysed by immunohistochemistry compared with our report wherein 100% serous EOCs were analysed by microarray, possibly attributing the discrepancy to multiple histological types. A common thread, however, is the potential of an alternate MMR-independent role of MLH1 in acquired cisplatin resistance, a mechanism still unclear (O'Brien and Brown, 2006).

A differential response of cells with proficient- and deficient-MLH1 in sensitivity to cisplatin and 6-TG in ovarian cells was observed in this study. The presence of wt MLH1 (OVCAR3) or a re-expression in an MLH1-defective A2780 variant did not significantly alter the proliferation rate in cisplatin response, but significantly increased sensitivity to 6-TG, a chemotherapeutic purine nucleoside analogue, the primary mechanism of action of which is dependent on the presence of a functional DNA MMR system (Hawn *et al*, 1995; Buermeyer *et al*, 1999; Yan *et al*, 2003) consistent with earlier reports (Aquilina *et al*, 2000; Branch *et al*, 2000). However, both 6-TG and cisplatin induced an MLH1-dependent apoptosis and arrest in the G2/M phase of cell cycle (our unpublished observations), consistent with earlier reports in OVCAR3 (Aquilina *et al*, 2000; Kolfschoten *et al*, 2002) (O'Brien and Brown, 2006). The MLH1 proteolysis in cells undergoing apoptosis correlated with the increased expression of active forms of caspase-3 and -9. Chen *et al* (2004), using ^35^S-labelled full-length MLH1 protein in an *in vitro* assay, have shown that MLH1 is cleaved at Asp418 specifically by caspase-3, leading to the partial translocation of MLH1 from the nucleus to the cytoplasm and the release of a pro-apoptotic C-terminal fragment, similar to the 44-kDa MLH1 fragment identified after cisplatin and 6-TG treatment in this study. These data suggest that ovarian cancer cells undergoing MLH1-dependent, chemotherapy-induced apoptosis signal XIAP destabilisation and release of active caspase-3. In this response, MLH1 appears functionally similar to other DNA damage-sensing proteins such as ICAD; cleavage of ICAD by caspase-3 generates a pro-apoptotic fragment that further increases apoptotic signalling (Tang and Kidd, 1998). Whether the cleaved MLH1 fragments remain associated as a heterodimer to increase apoptotic signalling is the subject of ongoing investigation.

A defect in caspase activation because of the expression of anti-apoptotic proteins such as XIAP is believed to be an important mechanism of acquired therapeutic resistance in multiple cancer models (Sasaki *et al*, 2000; Li *et al*, 2001; Mansouri *et al*, 2003; Amantana *et al*, 2004; Yang *et al*, 2005; Schimmer *et al*, 2006; Aird *et al*, 2008). It has been shown that the inability of cisplatin to induce apoptosis in EOC may be because of its inability to suppress XIAP with the corollary that XIAP may be overexpressed in cisplatin-resistant EOC. The XIAP inhibitors induce apoptosis by relieving its inhibition on caspases; however, Sasaki *et al* (2000) using an adenoviral antisense construct have reported that XIAP inhibition induced apoptosis only in wt-p53 cells (A2780 cisplatin sensitive and C13, cisplatin resistant) and not in p53-mutant/null cells (cisplatin resistant A2780cp or SKOV3). Similarities can be drawn as the C13 and A2780 cells are MLH1 proficient and the other two are MLH1 deficient. In addition, in this study, XIAP inhibition decreased cell viability and corresponded with MLH1 proteolysis only in MLH1 proficient although p53-mutant ovarian cancer cells and was ineffective in MLH1-deficient cells, revealing a potential p53-independent but MLH1-dependent effect. However, no significant synergy was observed using XIAP siRNA in combination with cisplatin, suggesting the possibility that the presence of both p53 and MLH1 would be critical for a potent response. Further studies testing various dosing regimens are necessary to evaluate whether any potential synergy exists between cisplatin and XIAP inhibition in MLH1-proficient ovarian cancer cells. Although, MLH1 proteolysis corresponded with a decrease in inactive procaspase-9 and an increase in caspase-3 in the cell-death response to XIAP inhibition, it would be important to determine whether MLH1 is indeed a direct substrate of caspases in cancer cells. As targeting XIAP is an attractive anticancer strategy (Schimmer *et al*, 2006), MLH1 expression may therefore be useful for predicting response to XIAP inhibitors alone or in combination with chemotherapy in EOC patients.
